# Feasibility of a walking virtual reality system for rehabilitation: objective and subjective parameters

**DOI:** 10.1186/s12984-016-0174-1

**Published:** 2016-08-09

**Authors:** Adrián Borrego, Jorge Latorre, Roberto Llorens, Mariano Alcañiz, Enrique Noé

**Affiliations:** 1 Neurorehabilitation and Brain Research Group, Instituto de Investigación e Innovación en Bioingeniería, Universitat Politècnica de València, Camino de Vera s/n, 46022 Valencia, Spain; 2 Servicio de Neurorrehabilitación y Daño Cerebral de los Hospitales NISA, Fundación Hospitales NISA, Río Tajo 1, 46011 Valencia, Spain

**Keywords:** Virtual reality, Walking, Motion tracking, Presence, Ecological validity

## Abstract

**Background:**

Even though virtual reality (VR) is increasingly used in rehabilitation, the implementation of walking navigation in VR still poses a technological challenge for current motion tracking systems. Different metaphors simulate locomotion without involving real gait kinematics, which can affect presence, orientation, spatial memory and cognition, and even performance. All these factors can dissuade their use in rehabilitation. We hypothesize that a marker-based head tracking solution would allow walking in VR with high sense of presence and without causing sickness. The objectives of this study were to determine the accuracy, the jitter, and the lag of the tracking system and its elicited sickness and presence in comparison of a CAVE system.

**Methods:**

The accuracy and the jitter around the working area at three different heights and the lag of the head tracking system were analyzed. In addition, 47 healthy subjects completed a search task that involved navigation in the walking VR system and in the CAVE system. Navigation was enabled by natural locomotion in the walking VR system and through a specific device in the CAVE system. An HMD was used as display in the walking VR system. After interacting with each system, subjects rated their sickness in a seven-point scale and their presence in the Slater-Usoh-Steed Questionnaire and a modified version of the Presence Questionnaire.

**Results:**

Better performance was registered at higher heights, where accuracy was less than 0.6 cm and the jitter was about 6 mm. The lag of the system was 120 ms. Participants reported that both systems caused similar low levels of sickness (about 2.4 over 7). However, ratings showed that the walking VR system elicited higher sense of presence than the CAVE system in both the Slater-Usoh-Steed Questionnaire (17.6 ± 0.3 vs 14.6 ± 0.6 over 21, respectively) and the modified Presence Questionnaire (107.4 ± 2.0 vs 93.5 ± 3.2 over 147, respectively).

**Conclusions:**

The marker-based solution provided accurate, robust, and fast head tracking to allow navigation in the VR system by walking without causing relevant sickness and promoting higher sense of presence than CAVE systems, thus enabling natural walking in full-scale environments, which can enhance the ecological validity of VR-based rehabilitation applications.

## Background

Virtual reality (VR) is an increasingly common term to refer to the real-time computer-generated simulation of a three-dimensional environment that replaces the natural sources of stimulation of the real world by artificial stimulation in different channels. The extent to which an individual is unable to acknowledge that an experience is computer-generated, known as the level of presence [1, 2], and other elicited subjective perceptions in VR are determined by user and media characteristics [3, 4]. User characteristics involve demographic aspects, motor or cognitive limitations, personality, and mood. Media characteristics involve not only the technological properties of the presentation of sensory information and the facilitation of the interaction, but also the content of the virtual environment (VE). The technology-mediated simulation of enriched environments and the provision of controlled sensory stimulation enable immersion in potentially hazardous and ecologically valid environments. These characteristics can be specially interesting for rehabilitation because they may allow to exceed the boundaries of the clinical setting and provide customized training to each participant [5, 6].

Interaction within VE has been facilitated through specific body movements [7, 8], finger touches [9, 10], or specific devices [5]. Even though different solutions have been successfully used to track upper [11] and lower limb movements [4, 8, 12], the implementation of walking navigation in VR still poses a technological challenge for current motion tracking systems, which are only capable of tracking a limited working area. Different metaphors have been proposed to simulate walking in VR applications. VR setups that involve computer screens or Cave automatic virtual environment (CAVE) systems, a cube composed of display-screens that completely surround the viewer [13], commonly use flysticks, gamepads, or 3D mice [5, 14]. These devices translate hand movements into displacements in the VE without involving real gait kinematics [14], limiting presence and affecting performance, which can even dissuade their use in rehabilitation. Gait training systems commonly incorporate treadmills while the VE is displayed in projectors [15–17] or TV screens [18, 19], showing real-world video-recording [16] or virtual scenarios [17–19]. However, besides its essential equivalence, only straight walking is allowed and kinematics of human walking have been reported to differ in treadmill and overground [20–22]. All of these factors must be taken into account because the way that sensory stimulation is provided and how interaction is facilitated regulate the capability of delivering an illusion of reality to the senses of a human participant [23], and also modulates the movement kinematics [24] and, in the end, the ecological validity of the simulation [25].

Real walking in VR has been recently enabled by an infrared camera-based motion tracking solution [26, 27], similar to those used in gait analysis laboratories [28]. Different cameras, arranged around the working area, estimate the position of a constellation of markers that are attached to a head mounted display (HMD), which provides visual feedback of the VR according to the head location and orientation. Since the working area covered by each camera is limited, a dedicated space with a remarkable number of cameras is needed, which increases the cost and the complexity of the system. A hybrid tracking solution that estimates the relative movement of both feet with inertial sensors and corrects the drift error from GPS data has been presented for outdoor walking navigation, using an HMD as a display [29]. However, the system provides visual stimulation without taking the position of the head into account, which can affect presence, balance, and even produce sickness [30], and estimates displacements from inertial sensors, which can produce relevant inaccuracies [31].

Fiducial markers are artificial landmarks added to the real world that enable accurate pose detection for applications ranging from augmented reality to robot navigation [32]. Marker-based algorithms obtain the camera pose from correspondences between specific features of the markers in the real world and their camera projections with high speed and accuracy [33]. We hypothesized that a marker-based head tracking solution would be accurate and robust enough to provide consistent head tracking in the VE, which, in turn, would enable navigation through walking in a room-size environment without causing sickness. Moreover, we conjecture that these factors would promote a stronger sense of presence than that elicited by laboratory-grade CAVE systems, which have been shown to provide higher levels of presence than HMD [14, 34]. Therefore, the objectives of this study were: first, to determine the accuracy, jitter, and the lag of a marker-based head tracking system, and second, to compare the elicited presence and sickness of a walking VR system with that of a CAVE system.

## Methods

A room-size walking VR system using a marker-based head tracking was designed and implemented to test the two hypotheses of the study, which were investigated in separate studies. The aim of the first study was to determine the objective parameters of the tracking system, defined by the accuracy, the jitter, and the lag. The aim of the second study was to compare the sickness and the subjective sense of presence elicited by the walking VR system and a CAVE system.

### Instrumentation

#### Walking VR system

**Fig. 1:**
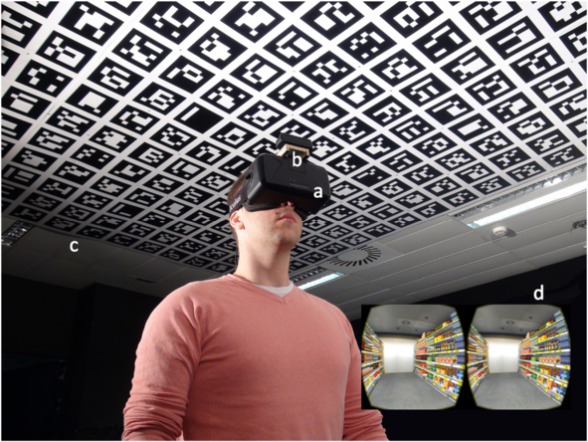
Setup of the walking virtual reality system. Experimental setup of the walking virtual reality system: **a** head mounted display; **b** RGB camera; **c** pattern of fiducial markers; and **d**) snapshot virtual environment that is being displayed to the user

The experimental system presented in this paper consisted of 1) an HMD, the Oculus DK2 (Oculus VR, Irvine, CA); 2) a RGB camera, the PlayStation®Eye Camera (Sony® Corporation, Tokyo, Japan) with an additional lens (4.3 mm, 70° FOV); 3) a pattern of markers fixed to the ceiling at 2.65 cm of height; 4) and a laptop (Fig. 1). The hardware components of the laptop included an 8-core Intel® Core™ i7 Haswell @ 2.50 GHz, 8 GB of RAM, and a NVIDIA® Geforce® GTX 860 M with 2GB of GDDR5.

The camera was fixed at the top of the HMD, pointing upwards. The additional lens was mounted on the camera to improve image quality. The camera was configured to capture standard video with a frame rate of 75 Hz at a 640 × 480 pixel resolution. The pattern of markers of the experimental setting consisted of 17 × 26 square fiducial markers, 18 × 18 cm each, separated 4 cm, thus covering an area of 3.78 × 5.76 m in the ceiling of the experimental room. However, wider areas can be covered using more markers (until 1024) or increasing their size. Four spotlights were placed in each corner of the room and were indirectly oriented to the ceiling to create homogeneous lighting conditions. The pattern of markers were generated using the ArUco library [33], a minimal library for augmented reality applications based on OpenCV. The library allowed to estimate the camera pose (position and rotation) from the correspondences between known points in the pattern of markers and their camera projections. Even though this library provides information of both position (x, y, and z coordinates) and orientation (yaw, pitch, and roll), only positional tracking was considered. The orientation of the head, and thus the orientation of the camera, was provided by the HMD.

It is important to highlight that even though the Oculus DK2 includes an external infrared camera to provide positional tracking, it is only capable of tracking short displacements of the head and users must be in front of the camera. Since the requirement of the system was to allow walking, this tracking capability was discarded.

#### CAVE system

**Fig. 2:**
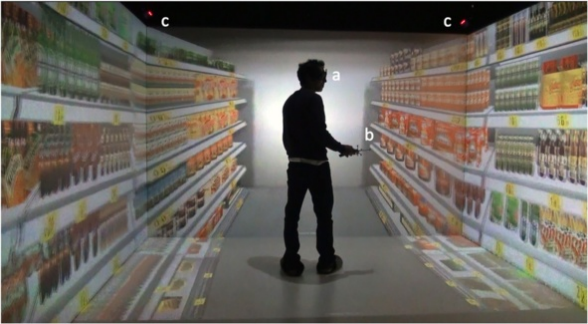
Setup of the CAVE system. Experimental setup of the CAVE system: **a** 3D glasses; **b** interaction device; and **c** infrared tracking cameras

The CAVE system consisted of 1) a conventional four-wall CAVE configuration (three vertical walls and floor), 2) four projectors F35 AS3D (Barco N.V., Poperinge, Belgium), one for each wall; 3) four infrared Trackpad cameras (Advanced Realtime Tracking GmbH, Munich, Germany) fixed to the upper frame of the vertical walls and pointing to the center of the CAVE; 4) a pair of 3D glasses Crystaleyes 3 (StereoGraphics, San Rafael, USA) with a constellation of infrared reflective markers; 5) a Flystick3 interaction device (Advanced Realtime Tracking GmbH, Munich, Germany), and 6) five high-end graphics computers, one master and four slaves connected through a high speed network (Fig. 2). The hardware components of the computers included an Intel® Xeon® CPU ES-2620 @ 2.00 GHz, 16 GB of RAM, and a NVIDIA® Quadro® 5000.

The walls were 350 cm wide and 204 cm long. The projectors back-projected stereoscopic images in each wall with a resolution of 1868 × 1200 pixels at 120 Hz. The infrared cameras tracked the 3D glasses, which provided information of the position and orientation of the head. The Flystick3 was used to navigate within the VE using a small joystick on the top of the device.

#### Virtual environment

**Fig. 3:**
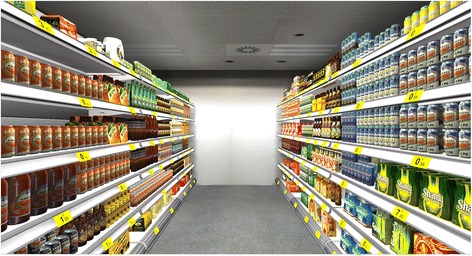
Virtual environment. A virtual aisle of a grocery store with 72 different kind of sodas was used in the experiment

The environment represented an aisle of a grocery store, defined by two shelves with different kind of sodas. The shelves were 4 m long and 2 m tall. The separation between them was 1.5 m. Each shelf consisted of six racks with 72 different items. The price of each soda was indicated in the shelf (Fig. 3). Users were not represented.

The VE was programmed using Unity 3D (Unity Technologies ApS, San Francisco, CA).

### Study 1: Objective parameters

#### Procedure

To estimate the accuracy and the jitter of the head tracking, a 6 × 10 grid with 50 cm × 50 cm squares was marked on the floor under the pattern of markers, covering an area of 15 m^2^. The RGB camera was fixed in all of the intersection points of the grid (77 in total) using a specifically designed device that prevented movement and allowed height adjustment. The position and the jitter at each point was estimated during 5 s at 3 different heights: 3 cm (on the floor), 1.3 m (approximate height of a subject’s head in sitting position), and 1.7 m (approximate height of a subject’s head in standing position). Since accuracy and jitter at a given height depended on the number of markers detected, this value was registered in the center of the grid at each height.

To estimate the relationship between the speed of the head and the number of markers detected, an experimenter equipped with the modified HMD walked in a straight line across the walking VR system at three different speeds. The starting and finishing line were marked on the floor. The speed of the head and the number of markers detected at each frame were recorded.

Finally, to estimate the lag of the system, the camera was attached to a stick and the experimenter violently swayed it and stop it for 20 times. Another PlayStation®Eye camera simultaneously recorded the movements of the experimenter and the display of the HMD at 75 Hz.

#### Data analysis

Accuracy (e) was estimated as the mean difference between the position of the camera measured in the real world and its estimated position. The jitter (j) was defined as the standard deviation in the estimated position in the time interval as follows:

$$ \begin{array}{l}e=\frac{1}{N}{\displaystyle \sum_{i=1}^N}\left|{X}_i-{\tilde{X}}_i\right|\hfill \\ {}j=\sqrt{{\displaystyle \sum_{i=1}^n}\frac{X_i^2}{N}-{\overline{X}}^2}\hfill \end{array} $$

where *N* is the number of measurements provided by the tracking system in 5 s; *X*_*i*_ is the real position of the camera; ${\tilde{X}}_i$ is the estimated position; and $\overline{X}$ is the mean position during these 5 s. Regarding jitter, the mean and the standard deviation for each coordinate were calculated [4].

The lag was estimated as the delay between the frame where the experimenter stopped the movement and the frame where the display of the HMD represented the end of the movement of the VE.

### Study 2: Subjective responses

#### Participants

Healthy subjects older than 18 years without motor or cognitive limitations were recruited for this study. Forty-seven participants (26 men and 21 women) were finally involved. Subjects had a mean age of 28.1 ± 5.3 years old, had an education of 22.1 ± 4.4 years, and rated their experience with videogames as 5.8 ± 3.3 over 10. All of the participants provided informed consent to participate in the study.

#### Procedure

Two experimenters were in charge of conducting the sessions, equipping the participants, and providing safety, guidance, and comfort. Participants wore the 3D glasses in the CAVE and the modified HMD in the walking VR system. Navigation was performed through the Flystick3 in the CAVE (small displacements in this environment, even possible, were not allowed) and by natural ambulation in the walking VR system. In this latter system an experimenter carried the laptop and handled the wires that connected the HMD and the camera to the computer to avoid tangles.

Before experimenting with each system, subjects were briefly introduced to the technology and were allowed to interact with them during 5 minutes. For this exploratory session, the same VE without items (the same aisle with empty racks) was used. After that, subjects were located in the center of the CAVE system or in one end of the walking VR system, and the experiment started. Participants, who were initially located in one end of the aisle in the VE, were required to find the price of five items. An experimenter asked participants for the price of the items consecutively and subjects had to explore the VE to find the items and tell the price. If the price was correct, the experimenter gave the description of the next item to be found. If the price was incorrect, the experimenter repeated the description of the current item. Participants had a maximum of 5 minutes to find all of the items. Subjects performed the task in both VR systems in counterbalanced order. Ten minute breaks were allowed between systems.

After each condition, participants rated the sickness or vertigo experienced during the virtual exposure in a seven point rating scale and were required to fill two questionnaires about presence. Assessment included the original Slater-Usoh-Steed Questionnaire [35] and a modified version of the Presence Questionnaire [36]. The Slater-Usoh-Steed Questionnaire consists of three items rated on a seven point rating scale, that assess the sense of being in the virtual environment, the extent to which the VE becomes real, and the extent to which the VE is thought of as a place visited. Scores to this questionnaire range from 3 to 21. The modified version of the Presence Questionnaire consisted of 21 items rated on a seven point rating scale that assessed presence taking into account the influence of the visual aspects, interaction, consistency with the real world, and subjective factors (Table 1). Scores to this questionnaire range from 21 to 140.

**Table 1:** Modified presence questionnaire

Questionnaire
Visual aspects 1. How much did the visual aspects of the environment involve you? 2. How aware were you of your display devices? 3. How completely were you able to actively survey or search the environment using vision? 4. How closely were you able to examine objects? 5. How well could you examine objects from multiple viewpoints? 6. How much delay did you experience between your actions and expected outcomes? 7. How much did the visual display quality interfere or distract you from performing assigned tasks or required activities?
Interaction 8. How natural did your interactions with the environment seem? 9. How natural was the mechanism which controlled movement through the environment? 10. How aware were you of your control devices? 11. How compelling was your sense of moving around inside the virtual environment? 12. How distracting was the control mechanism? 13. How much did the control devices interfere with the performance of assigned tasks or with other activities?
Consistency with the real world 14. How responsive was the environment to actions that you initiated (or performed)? 15. How much did your experiences in the virtual environment seem consistent with your real-world experiences? 16. Were you able to anticipate what would happen next in response to the actions that you performed?
Subjective factors 17. How much were you able to control events? 18. How involved were you in the virtual environment experience? 19. How quickly did you adjust to the virtual environment experience? 20. How proficient in moving and interacting with the virtual environment did you feel at the end of the experience? 21. Did you learn new techniques that enabled you to improve your performance?

Modified version of the Presence Questionnaire. Items were categorized in four domains: visual aspects, interaction, consistency with the real world, and subjective factors

#### Data analysis

Scores to the questionnaires were compared with independent sample t-tests. The α level was set at 0.05 for all analyses (two-sided). All analyses were computed with SPSS for Windows®, version 22 (IBM®, Armonk, NY, USA). Investigators performing the data analysis were blinded.

## Results

### Study 1: Objective parameters

Both accuracy and jitter proved to be dependent on the height (Table 2). Higher accuracy (lower error) and lower jitter were registered at 1.3 and 1.7 m. In contrast, worse results were obtained on the floor. Interestingly, these parameters did not depend on the other spatial coordinates. Values remained almost invariant for a given height.

**Table 2:** Objective parameters

	On the floor (0 m)	In sitting position (1.3 m)	In standing position (1.7 m)
Number of markers (*n*)	68	15	7
Accuracy (cm)			
X coordinate Y coordinate Z coordinate Total	0.83 ± 0.42 0.73 ± 0.41 0.87 ± 0.88 0.81 ± 0.61	0.57 ± 0.26 0.55 ± 0.27 0.58 ± 0.27 0.57 ± 0.27	0.51 ± 0.26 0.54 ± 0.27 0.57 ± 0.26 0.54 ± 0.26
Jitter (cm)			
X coordinate Y coordinate Z coordinate Total	0.32 ± 0.53 0.24 ± 0.51 0.04 ± 0.09 0.21 ± 0.44	0.05 ± 0.08 0.05 ± 0.09 0.03 ± 0.04 0.05 ± 0.07	0.07 ± 0.13 0.06 ± 0.14 0.05 ± 0.06 0.06 ± 0.11

Number of the markers detected in the center of the grid. Accuracy and jitter values estimated in the intersection points of the grid. Results are defined in terms of mean and standard deviation

**Fig. 4:**
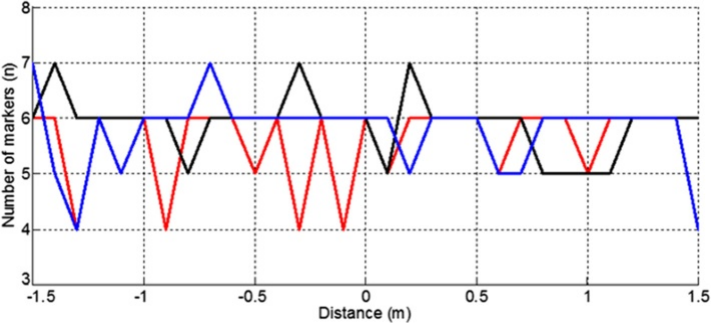
Number of markers detected at different walking speed. Number of markers detected each 10 cm at three different speeds: 0.38 m/s (red), 1.01 m/s (blue), and 1.79 m/s (black)

Likewise, the number of markers detected was also dependent on the height (Table 2), but, in contrast, this value proved to be not very dependent on the walking speed (Fig. 4).

Finally, the lag of the system was shown to be 120 ms (nine frames at 75 Hz) in all the repetitions.

### Study 2: Subjective responses

All of the participants finished the experiment and reported that the experience with the systems did not cause relevant levels of sickness. The walking VR system caused slightly higher sickness (2.4 ± 0.6) than the CAVE (2.2 ± 0.7), but no statistical significance was found between the ratings (*p* = 0.641).

In contrast, results showed significant differences in the sense of presence elicited by both systems (Table 3). Participants reported to have experienced higher presence in the walking VR system than in the CAVE in both the Slater-Usoh-Steed Questionnaire (17.6 ± 0.4 vs 14.6 ± 0.6) and the Presence Questionnaire (107.8 ± 2.0 vs 93.5 ± 3.2). A more in depth analysis showed that both systems were reported to have similar visual characteristics but significantly different interaction mechanisms, which elicited the greatest difference between systems. Participants also reported that the experience elicited different subjective factors and different perception of the consistency with the real world.

**Table 3:** Subjective responses

	CAVE	Walking virtual reality system	Significance
Slater-Usoh-Steed Questionnaire (3–21)	14.6 ± 0.6	17.6 ± 0.3	*p* = 0.000
Presence Questionnaire (21–147)	93.5 ± 3.2	107.4 ± 2.0	*p* = 0.000
Visual aspects (1–7)	5.2 ± 1.1	5.3 ± 0.8	NS
Interaction (1–7)	4.4 ± 1.3	5.5 ± 0.8	*p* = 0.000
Consistency (1–7)	5.1 ± 1.2	5.8 ± 0.8	*p* = 0.001
Subjective factors (1–7)	5.1 ± 1.2	5.6 ± 0.7	*p* = 0.001

Sense of presence assessed with the Slater-Usoh-Steed Questionnaire and the Presence Questionnaire elicited in both the CAVE and the walking system. Results are defined in terms of mean and standard deviation. NS: non significant

## Discussion

This paper presents an experimental VR system that enables head tracking through a fiducial marker-based solution, accurate, robust, and fast enough to allow natural navigation in the VE by walking in the real world without causing relevant sickness or vertigo. The system has been shown to elicit significantly higher sense of presence than CAVE systems.

Fiducial marker tracking has been extensively used in augmented reality applications to estimate the camera pose with high accuracy and low computational cost, thus allowing to add virtual elements with adequate location and orientation in real time [33, 37, 38]. Different studies have determined the accuracy in the position estimation from a single marker. Reported errors vary from 11 cm [39] to 1.4 cm at 1 m [40]. The use of a more reliable tracking library in combination with multiple markers, which defined an overdetermined system of equations, could explain the higher accuracy achieved by our solution, even at larger distances [33]. In accordance with previous reports [39, 40], not only accuracy but also jitter were distance dependent. The worst results were achieved on the floor, while estimations at heights simulating the sitting and standing position were very similar. It is important to highlight that jitter values at those heights were less than 1 mm, which can be considered jitterless considering that it was imperceptible while wearing the HMD. Far from being still, the head is continuously stabilized in space to provide a steady reference, not only in standing but also during walking [41], which could have made impossible to differentiate between the natural head sway from that caused by the jitter of the tracking system. Our results could evidence a trade-off between distance to the markers and the number of markers taken into account for the calculation. Interestingly, the number of markers proved to be almost not dependent on the speed of the head. Even at faster speeds than the considered comfortable walking speed (0.9–1.3 m/s) [42], a minimum of four markers were detected in every moment. With regards to the lag of the system, experimental results showed values slightly higher than, according to Bloch's Law [43], the minimum duration that visual stimuli should last to be physiologically detected with independence of their intensity. It is also important to highlight that the lag of the system was shown to be smaller than that reported to cause sickness [44, 45]. These results should be also emphasized since delayed visual feedback has been shown to affect performance, but not so much for delays of 120 ms [46].

The low jitter values and lag can be specially relevant because visual manipulation in VR can cause a sensory mismatch that has been shown to produce postural instability. Visual-vestibular conflict produced by rotating the world around the head has been shown to increase spatial disorientation [47]. Furthermore, the direction and velocity of visual flow in the VE modulate the postural reorienting responses in the real world [48], which, in turn, have been shown to be dependent on visual stimuli that are specific to the display device [49]. In addition, sensory mismatch has been reported to be an important cause of sickness in VR [44, 50, 51]. The absence of serious reports of sickness or vertigo in our study could also highlight the performance of the tracking system.

In contrast to our study, previous reports have shown that CAVE systems facilitate greater sense of presence than HMDs [14, 34, 52, 53]. The technological advances of the current HMDs and the natural navigation in the walking VR system could have promoted higher sense of presence in the walking VR system, which could justify the differences regarding interaction and consistency. Also supporting this, participants reported that visual aspects were similar in both systems but navigation caused the strongest differences. Interestingly, the match between proprioceptive information from human body movements and computer-generated sensory stimulation has been reported to modulate presence in VEs [14]. Our results are also in accordance with the role of immersion in presence, which is expected to contribute to increasing this sense [3, 54–56].

All these findings can be specially interesting for VR-based rehabilitation applications that involve navigation, because the solution presented here allows to replace navigation metaphors based on upper-limb movements [57] or joysticks [5] by natural walking in the real world, which can have special implications on spatial and visual memory, orientation, and spatial cognition [58]. It is important to highlight that the tracking area can be resized to fit wider working areas preserving the same accuracy and jitter characteristics, which could enable street-crossing or shopping in full-scale, thus enhancing the ecological validity of the simulation [25].

Limitations of this study must be taken into account. First, the characteristics of the sample, healthy young adults, could limit extrapolation of the results to other populations. Second, the visual estimation of the lag may be subject to errors. In addition, the different sources of the lag were not differentiated. Third, no avatar was used to represent the participants in the VE, which has been shown to modulate subjective factors in VR [59–61]. Fourth, even significant, the clinical relevance of the differences detected in both environments is unknown. Even though different attempts have been made to determine objective correlates of the sense of presence [62, 63], the subjectivity of the sense makes its assessment a challenge for researchers. Finally, the weight of the wearable devices and the particular characteristics of the visual display could alter the gait kinematics while walking in the VR system. Further research should address these issues.

However, the high performance of the head tracking, which provided accurate and robust location of the head and imperceptible lag, and the visual stimulation provided by a last-generation HMD allowed natural locomotion in VR without causing remarkable sickness and promoting higher sense of presence. These characteristics, together with the modularity of the system, enable natural walking in full-scale VEs, which can enhance the ecological validity of VR-based rehabilitation applications.

## Conclusions

This paper presents an experimental VR system that enables head tracking through a fiducial marker-based solution, accurate, robust, and fast enough to allow navigation in the VE by walking in the real world without causing relevant sickness or vertigo and promoting higher sense of presence than CAVE systems. These characteristics, together with the modularity of the system, enable natural walking in full-scale VEs, which can enhance the ecological validity of VR-based rehabilitation applications.

## Abbreviations

CAVE, cave automatic virtual environment; HMD, head mounted display; VE, virtual environment; VR, virtual reality
